# Prevalence and concordance of oral and genital HPV by sexual orientation among US men

**DOI:** 10.1093/jncics/pkac088

**Published:** 2022-12-15

**Authors:** Kalyani Sonawane, Shiang Shiuan Shyu, Haluk Damgacioglu, Ruosha Li, Alan G Nyitray, Ashish A Deshmukh

**Affiliations:** Department of Public Health Sciences, College of Medicine, Medical University of South Carolina, Charleston, SC, USA; Medical University of South Carolina Hollings Cancer Center, Charleston, SC, USA; Department of Biostatistics and Data Science, UTHealth School of Public Health, Houston, TX, USA; Department of Public Health Sciences, College of Medicine, Medical University of South Carolina, Charleston, SC, USA; Medical University of South Carolina Hollings Cancer Center, Charleston, SC, USA; Department of Biostatistics and Data Science, UTHealth School of Public Health, Houston, TX, USA; Clinical Cancer Center, Medical College of Wisconsin, Milwaukee, WI, USA; Center for AIDS Intervention Research, Medical College of Wisconsin, Milwaukee, WI, USA; Department of Public Health Sciences, College of Medicine, Medical University of South Carolina, Charleston, SC, USA; Medical University of South Carolina Hollings Cancer Center, Charleston, SC, USA

## Abstract

The objective of our study was to describe oral and genital human papillomavirus (HPV) infection prevalence and concordance by sexual orientation among US men using a nationally representative sample. We conducted a retrospective cross-sectional analysis of the 2013-2016 National Health and Nutrition Examination Survey. The survey conducts a physical examination and collects oral rinse and genital swab specimens; demographic and health behaviors are self-reported. We used descriptive statistics and multivariate regression models to estimate HPV infection prevalence and the likelihood of HPV infection, respectively. All analyses were adjusted for National Health and Nutrition Examination Survey design and weights, and statistical significance was tested at a 2-sided *P* value of less than .05. Men who have sex with men had a statistically significantly higher prevalence of oral HPV (high-risk, 9-valent, 4-valent, and HPV 16 and 18), genital HPV (9-valent, 4-valent, and HPV 16 and 18), and concordant oral and genital HPV (high-risk and 9-valent) infections compared with heterosexual men. Improved HPV prevention among men is needed.

The incidence of human papillomavirus (HPV)–associated cancers, particularly oropharyngeal and anal cancers, is rising rapidly among US men (1-4). Gay, bisexual, and other men who have sex with men, collectively referred to as MSM, represent a large (nearly 7 million) and growing population at elevated risk of developing anogenital and oral HPV-associated cancers (5-7). The study of HPV infection occurring at oral and genital sites among MSM and the extent to which HPV infection risk is elevated compared with heterosexual men is crucial. Furthermore, given the likelihood of HPV transmission that may occur between oral and genital sites, the study of the risk of genital and oral HPV infection concordance (ie, presence of the same HPV genotype at oral and genital sites) among MSM compared with heterosexual men is also important, although it is yet to be quantified. This study aims to describe oral and genital HPV infection prevalence and concordance by sexual orientation among US men.

We conducted a retrospective cross-sectional analysis of the 2013-2016 National Health and Nutrition Examination Survey (NHANES), a nationally representative survey of noninstitutionalized individuals in the United States. A clustered multistage probability sample of participants is identified, and information on sociodemographic and health and sexual behaviors is collected by trained interviewers during the home interview or in mobile examination centers. The survey also conducts a physical examination and collects oral rinse and genital swab specimens in the mobile examination centers. The oral rinse sample (10 mL sample of mouthwash or saline) and self-collected external genital swabs are refrigerated at 4°C and shipped by the NHANES personnel to a laboratory to perform polymerase chain reaction assay for genotyping. Details regarding the survey are available on the NHANES website (8).

In the current study, men aged 18-59 years of age with nonmissing data on sexual orientation and oral and/or genital HPV tests were identified. Sexual orientation was self-reported (male sex partners contact and sexual identity). The HPV genotypes were identified from assay results and classified (as per prior studies) as type 16, type 18, 4-valent vaccine types (HPV 6, 11, 16, 18); 9-valent vaccine types (HPV 6, 11, 16, 18, 31, 33, 45, 52, 58); and any high-risk types (HPV 16, 18, 26, 31, 33, 35, 39, 45, 51, 52, 53, 56, 58, 59, 66, 68, 73, 82) (9,10). Concordant infection was defined as the detection of the same HPV genotype at oral and genital sites. The difference in infection prevalence was examined using the Rao-Scott χ^2^ test. Multivariable logistic regression models, adjusted for age, race, and ethnicity (Black, Hispanic, Other, and White); cigarette use; lifetime number of sex partners; history of sexually transmitted infections; age at first sex; and circumcision, estimated the odds ratio for HPV infection and concordant infection. Statistical significance was tested at a 2-sided *P* value less than .05. All analyses were performed with SAS software version 9.4 (SAS Institute, Cary, NC, USA) using SAS PROC SURVEY procedures to incorporate sampling weights and to adjust for the complex survey design. The study was deemed exempt from review by the institutional review board committee as it utilizes deidentified publicly available dataset.

The final sample included 3232 (156 MSM and 3076 heterosexual) men with information on oral HPV infection, 2954 (113 MSM and 2841 heterosexual) men with data on genital HPV infection, and 2883 (109 MSM and 2774 heterosexual) men with information on both infections (Supplementary Table 1, available online). Prevalence of oral HPV infection was statistically significantly higher among MSM compared with heterosexual men—any high-risk (12.0% vs 6.2%; *P *=* *.009), 9-valent types (9.5% vs 2.9%; *P* < .001), 4-valent (8.2% vs 1.6%; *P* < .001), and type 16 and 18 (6.9% vs 1.3%; *P* < .001) (Table 1**)**. Prevalence of genital 9-valent (24.4% vs 13.3%; *P* = .006), 4-valent (19.0% vs 7.2%; *P *=* *.001), and type 16 and 18 (14.3% vs 5.0%; *P *=* *.01) HPV types was also statistically significantly higher among MSM compared with heterosexual men. Concordant oral and genital infection prevalence of any high-risk (5.5% vs. 1.4%; *P* < .001) and 9-valent (2.5% vs 0.4%; *P *=* *.07) HPV types was more common among MSM compared with heterosexual males. The likelihood of oral and genital any high-risk, 9-valent, 4-valent, and types 16 and 18 HPV infection was statistically significantly higher for MSM compared with heterosexual men (Supplementary Table 2, available online). The likelihood of high-risk oral and genital HPV concordance was statistically significantly greater for MSM compared with heterosexual men.

**Table 1.** Prevalence and concordance of oral and genital HPV infection among US men by sexual orientation, NHANES 2013-2016

HPV types	Oral HPV			Genital HPV			Oral- and genital HPV concordance^a^		
	MSM	Heterosexual	*P* ^b^	MSM	Heterosexual	*P* ^b^	MSM	Heterosexual	*P* ^b^
	(n = 156)	(n = 3076)		(n = 113)	(n = 2841)		(n = 109)	(n = 2774)	
HPV types 16, 18, 26, 31, 33, 35, 39, 45, 51, 52, 53, 56, 58, 59, 66, 68, 73, 82 (any high risk)									
Unweighted, with infection, No.	19	189	.009	35	795	.63	6	44	<.001
Prevalence (95% CI), %	12 (5.6 to 18.3)	6.2 (5.0 to 7.4)		30.1 (19.1 to 41.1)	27.6 (24.9 to 30.3)		5.5 (0 to 11.4)	1.4 (0.9 to 1.9)	
Weighted, with infection/total population, No.^c^	491 395/4 103 015	4 401 882/71 090 232		951 862/3 160 066	18 111 189/65 614 189		167 187/3 020 778	657 335/64 089 450	
HPV types 6, 11, 16, 18, 31, 33, 45, 52, 58 (9 valent)									
Unweighted, with infection, No.	11	86	<.001	27	405	.006	4	16	.007
Prevalence (95% CI), %	9.5 (3.7 to 15.3)	2.9 (2.2 to 3.6)		24.4 (14.5 to 34.3)	13.3 (11.6 to 15.0)		2.5 (0 to 5.7)	0.4 (0.2 to 0.7)	
Weighted, with infection/total population, No.^c^	389 535/4 103 015	2 078 201/71 090 232		770 065/3 160 066	8 732 472/65 614 189		74 921/3 020 778	278 506/64 089 450	
HPV types 6, 11, 16, 18 (4 valent)									
Unweighted, with infection, No.	9	55	<.001	19	214	.001	2	13	.22
Prevalence (95% CI), %	8.2 (2.6 to 13.8)	1.6 (1.2 to 2.0)		19.0 (8.7 to 29.2)	7.2 (6.1 to 8.4)		0.7 (0 to 1.9)	0.3 (0.1 to 0.4)	
Weighted, with infection/total population, No.^c^	336 024/4 103 015	1 126 245/71 090 232		599 461/3 160 066	4 753 939/65 614 189		21 410/3 020 778	169 349/64 089 450	
HPV types 16, 18									
Unweighted, with infection, No.	8	42	<.001	14	139	.01	2	10	.12
Prevalence (95% CI), %	6.9 (2.0 to 11.7)	1.3 (0.9 to 1.7)		14.3 (3.4 to 25.2)	5.0 (4.0 to 6.0)		0.7 (0 to 1.9)	0.2 (0.1 to 0.3)	
Weighted, with infection/total population, No.^c^	281 970/4 103 015	928 793/71 090 232		451 593/3 160 066	3 295 664/65 614 189		21 410/3 020 778	134 225/64 089 450	

a Concordant infection was defined as the detection of the same genotype of the HPV present at oral and genital sites. CI = confidence interval; HPV = human papillomavirus; MSM = men who have sex with men; NHANES = National Health and Nutrition Examination Survey.

b *P* value for Rao-Scott χ^2^ test accounting for survey design and adjusted for weights.

c Weighted No. and total population represent the number of men with infection and the total population in the United States, respectively, estimated using the NHANES sampling weights.

This study documents a higher prevalence of oral and genital HPV infection among MSM compared with heterosexual men. Our study further reports for the first time that concordant oral and genital HPV (high-risk and 9-valent) infection prevalence is also greater among MSM compared with heterosexual men. A previous study reported higher genital HPV prevalence among men having sex with men and women than among MSM or heterosexual men (11). A prior NHANES study also reported greater high-risk oral HPV infection prevalence among MSM compared with heterosexual men (12). Consistent with these studies, we found a higher prevalence of 9-valent, 4-valent, and type 16 and 18 oral and genital HPV infection and higher genotypes-specific concordance among MSM. Greater concordance of oral and genital HPV suggests that the transmission of genital and oral HPV may be more likely among MSM, possibly from autoinoculation by individuals through fingers or greater transmission risk through partners. Further research is needed to understand the factors that increase the susceptibility of HPV infection among MSM and the contribution of bidirectional transmission to cancer risk among MSM and heterosexual males. Careful consideration of the self-reported nature of the data and the limited sample for concordance analysis should be given when interpreting the findings of this study. The cross-sectional nature of our analysis also precludes causal inferences.

Our study has important cancer prevention and policy implications. The higher prevalence of oral and genital HPV among MSM underscores the importance of intensive efforts for HPV prevention. Unlike heterosexual males, the MSM population may not benefit from herd protection through female HPV vaccination, which increases the importance of achieving high vaccination coverage among males. Studies have documented that HPV vaccination is effective for protection against anogenital and oral HPV among MSM, particularly if initiated at younger ages (13,14). Unfortunately, HPV vaccination coverage among adolescent and young adult males is lower compared with females (15,16). Furthermore, only 32.8% of MSM aged 18-26 years were reportedly vaccinated in 2017 (17). Continued efforts are needed to improve vaccination coverage among adolescent and young adult boys and the currently unvaccinated MSM population to reduce their risk of developing HPV-associated cancers in future decades.

## Supplementary Material

pkac088_Supplementary_Data

## Data Availability

Data is available publicly on the NHANES website.

## Notes

**Role of the funder:** The funder had no role in the design of the study; collection, analysis, or interpretation of the data; the preparation of the manuscript or decision to submit it for publication.

**Disclosures:** Dr Sonawane has received consulting fees from Value Analytics Labs on unrelated projects. Dr Deshmukh has received consulting fees from Merck on unrelated projects. The remaining authors have no disclosures.

**Author contributions:** KS (Conceptualization, Formal Analysis, Funding acquisition, Resources, Supervision, Writing-original draft, Writing- reviewing & editing); SSS (Data Curation, Formal Analysis, Visualization); HD (Validation, Writing-original draft, Writing- reviewing & editing); RL (Formal Analysis, Visualization, Validation); AGN (Conceptualization, Writing-original draft, Writing- reviewing & editing); AAD (Conceptualization, Resources, Funding acquisition, Writing-original draft, Writing- reviewing & editing).

**Acknowledgements:** We acknowledge the National Center for Health Statistics (NCHS) of the Centers for Disease Control and Prevention (CDC) for making the National Health and Nutrition Examination Survey (NHANES) publicly available and men who participated in the survey.
