# Supplementary material for: Prevalence and concordance of oral and genital HPV by sexual orientation among US men
Source: JNCI Cancer Spectr. 2022 Dec 15;7(1):pkac088. doi: 10.1093/jncics/pkac088 (PMC9825748; doi:10.1093/jncics/pkac088)
Supplement: pkac088_Supplementary_Data [file pkac088_supplementary_data.pdf]

SUPPLEMENTARY MATERIAL

Prevalence and concordance of human papillomavirus infection by sexual orientation among US men

Authors: Kalyani Sonawane, PhD Shiang Shiuan Shyu, BS Haluk Damgacioglu, PhD Ruosha Li, PhD, Alan G. Nyitray, PhD Ashish A. Deshmukh, PhD, MPH

SUPPLEMENTARY TABLE 1: Characteristics of US men by sexual orientation, NHANES 2013-2016.

	MSM (n= 156)	Heterosexual (n= 3076)	P*
Age (Mean, SD)	38.2 (0.28)	44.6 (1.6)	<.001
Race/Ethnicity, n (%)			0.0012
Black	40 (9.9)	640 (11.3)	
Hispanic	13 (3.3)	289 (6.5)	
Other*	32 (10.9)	1002 (20.0)	
White	71 (76.0)	1145 (62.3)	
Cigarette use, n (%)			0.84
Ever/former	70 (65.4)	1701 (70.89)	
1-10 cigarettes/day	28 (19.2)	501 (17.05)	
11-20 cigarettes/day	11 (11.9)	231 (9.51)	
>20 cigarettes/day	5 (3.5)	46 (2.56)	
Number of sex partners, n (%)			0.09
0-1	31 (20.1)	559 (16.6)	
2-5	53 (34.4)	823 (26.7)	
6-10	20 (13.0)	621 (21.2)	
11-15	12 (7.8)	304 (11.3)	
≥16	38 (24.7)	757 (24.2)	

Abbreviations: HPV, human papillomavirus; NHANES, National Health and Nutrition Examination Survey; MSM, men who have sex with men
**P* values for *t* test (continuous variable) and chi-square test (categorical variables) adjusted for survey weights and design
**'Other' includes all individuals who identified themselves as 'other race' including multiracial.

SUPPLEMENTARY TABLE 2: Odds ratio for oral and genital HPV infection and concordance of oral-genital HPV infection among US men.

	Oral HPV OR (95% CI)**	Genital HPV OR (95% CI)**	Concordant oral-genital HPV* OR (95% CI)**
HPV types 16/18/26/31/33/35/39/45/51/52/53/56/58/59/66/68/73/82 (any high-risk)			
MSM	3.69 (1.32-10.35)	3.69 (1.51-9.07)	14.17 (4.01, 50.1)
Heterosexual	(reference)	(reference)	(reference)
HPV types 6,11,16,18,33, 45, 52, 58 (9-valent)			
MSM	5.06 (1.71, 15.03)	4.11 (1.44, 11.74)	21.71 (3.60, 130.96)†
Heterosexual	(reference)	(reference)	(reference)
HPV types 6,11,16,18 (4-valent)			
MSM	5.14 (1.91, 13.87)	5.18 (1.74, 15.45)	2.76 (0.48, 15.99)†
Heterosexual	(reference)	(reference)	(reference)
HPV type 16/18			
MSM	6.84 (2.36, 19.88)	6.26 (1.94, 20.23)	4.42 (0.88, 22.16)†
Heterosexual	(reference)	(reference)	(reference)

Abbreviations: CI = confidence interval; HPV, human papillomavirus; NHANES, National Health and Nutrition Examination Survey; MSM, men who have sex with men; OR = odds ratio.
*Estimates may not be reliable due to low sample size.
**Concordant infection was defined as the detection of the same genotype of the HPV present at both oral and genital sites.
†Odds ratio from logistic regression models adjusted for age, race/ethnicity, cigarette use, lifetime number of sex partners, history of sexually transmitted infections, age at first sex, and circumcision.
